# Progress, Challenges, and Standardization Pathways in the Isolation and Purification Techniques of Plant-Derived Vesicles

**DOI:** 10.3390/plants15152292

**Published:** 2026-07-27

**Authors:** Junle Lv, Samia Muhammad Arif, Ruonan Que, Lin Zhang, Bingxian Yang

**Affiliations:** College of Life Sciences and Medicine, Zhejiang Sci-Tech University, Hangzhou 310018, China; junle_lv@163.com (J.L.); 20251109e1002@mails.zstu.edu.cn (S.M.A.); 2023332864041@mails.zstu.edu.cn (R.Q.)

**Keywords:** plant-derived vesicles (PDVs), isolation and purification technology, methodological advances, technical challenges, standardization pathways

## Abstract

Plant-Derived Vesicles (PDVs) are natural bioactive nanostructures and potential carriers that hold significant promise for applications in biotherapy and drug delivery. However, their isolation and purification technologies face numerous challenges, including the physical barriers posed by plant cell walls and interference from secondary metabolites. This makes it difficult for existing technologies to achieve both scalable production and high purity. This review evaluates mainstream isolation methods based on six dimensions: operational difficulty, cost, throughput, purity, yield, and impact on vesicle integrity. It also proposes a plant organ-based isolation strategy. Furthermore, this review explores standardized characterisation and quality control strategies for plant exosomes, aiming to provide a standardized and operational technical framework for research in this field. Future research will require standardized production, robust quality control frameworks, and clear elucidation of the action mechanism to promote the clinical application of PDVs.

## 1. Introduction

### 1.1. PDVs and Research Progress

Extracellular vesicles (EVs) are lipid bilayer-enclosed particles naturally released from cells, with diameters ranging from approximately 40 to 1000 nm. The term EVs encompasses, but is not limited to, exosomes, microvesicles, and apoptotic bodies [1]. Recent studies have shown that almost all living cells can secrete EVs, including plants, animals, and microorganisms [2,3]. By transporting proteins, lipids, nucleic acids, and other bioactive molecules between cells, EVs serve as key mediators of intercellular communication and regulate a wide range of physiological processes [4].

Exosomes are the most extensively studied subtype of EVs because of their pivotal roles in intercellular communication and their considerable potential in disease diagnosis, drug delivery, and regenerative medicine. Although EVs are widely distributed in plants, animals, and microorganisms, early research on exosomes primarily focused on animal exosomes. The research history of animal exosomes dates back to the 1980s. In 1983, Johnstone’s team first observed a vesicle structure with a diameter of approximately 50 nm in the supernatant of a sheep reticulocyte culture, and officially named it an “exosome” in 1987 [5]. Since then, research in this field has continued to deepen. Valadi et al. [6] first demonstrated that exosomes carry functional mRNA and miRNA and can be taken up by recipient cells, thereby regulating the expression of target genes. This discovery highlighted the crucial role of exosomes as intercellular information carriers, thereby promoting their investigation as potential components in drug delivery systems. In the past five years, the application of exosomal miRNA in disease diagnosis and treatment has reached its peak, especially in the fields of tumour microenvironment regulation, immune escape, and neurodegenerative diseases [7]. Research in this field became more standardized and comprehensive with the establishment of the International Society for Extracellular Vesicles (ISEV) in 2011. The Nobel Prize recognition of the vesicle transport mechanism in 2013 further promoted the application of exosomes in biomedicine.

Plant cells have also been shown to secrete extracellular vesicles that are morphologically and biogenetically comparable to animal exosomes. These vesicles are commonly referred to as exosome-like nanoparticles (ELNs) because of their structural and functional similarities to mammalian exosomes. However, owing to the lack of consensus definitions, consistent nomenclature, and standardized practices for biologically derived vesicles from plants, largely resulting from the absence of well-defined biogenetic pathways and rigorous physicochemical characterization, no universally accepted terminology currently exists. Therefore, these vesicles are collectively referred to as plant-derived vesicles (PDVs) throughout this review. PDVs were first observed in carrot (*Daucus carota* subsp. *sativus*) cells in 1965 and were termed “secondary vesicles” [8]. However, systematic research remained limited for a long time because of the physical barriers imposed by plant cell walls and the lack of specific molecular markers. Their existence was subsequently confirmed following their isolation from tomato (*Solanum lycopersicum*) cells and sunflower (*Helianthus annuus*) exudates in 2008 and 2009, respectively [9]. A major breakthrough came in 2013 when PDVs with diameters of 50–300 nm were successfully isolated from grape (*Vitis vinifera*) juice. Their high yield, ease of isolation, and remarkable biological activity attracted widespread attention and greatly accelerated subsequent research on PDVs [10].

**Figure 1:**
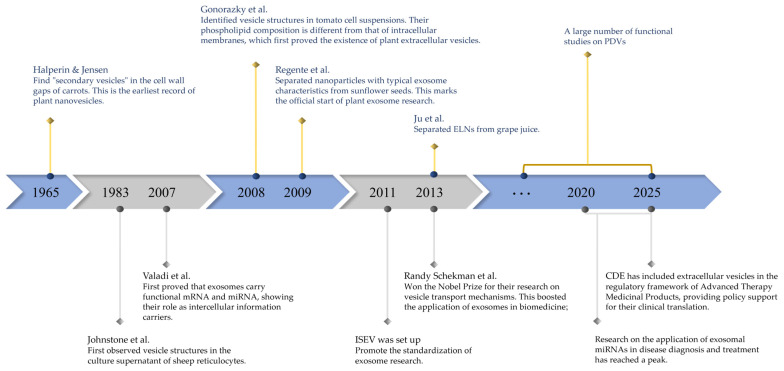
Timeline of key milestones in the evolution of extracellular vesicle research, highlighting the emergence and development of PDV research. The timeline summarizes the chronological evolution of the extracellular vesicle field. Milestones positioned above the timeline represent key developments that specifically advanced PDV research, whereas milestones positioned below the timeline indicate major advances in the broader exosome field. Blue and gray timeline segments are used to visually distinguish these two categories. Because important developments in PDV research often occurred alongside advances in the broader exosome field, the colored timeline segments may overlap or alternate during certain periods [5,6,8,9,10,14,15].

With advancements in separation technology, research on PDVs has grown rapidly, especially in disease treatment. Many studies focus on plants, considering exosomes as messengers for cell-to-cell communication and confirming their roles through cell-based assays. Raimondo et al. [11] discovered that nanoscale vesicles in lemon (*Citrus* × *limon*) juice can inhibit chronic myeloid leukemia tumor growth by activating TRAIL-mediated apoptosis. Kim et al. [12] found that ginseng (*Panax ginseng*)-derived ELNs can effectively reduce colitis symptoms in mouse models, showing potential for treating inflammatory bowel disease by suppressing pro-inflammatory cytokines and regulating gut microbiota. Likewise, pomegranate (*Punica granatum* L.)-derived ELNs accumulate in the liver after oral intake and effectively alleviate high-fat diet-induced non-alcoholic fatty liver disease by improving mitochondrial function, increasing ATP production, boosting mitochondrial complex I activity, and lowering oxidative stress [13]. In June 2025, the China Medical Products Administration included EVs in the regulatory framework for Advanced Therapeutic Drugs, supporting the clinical translation of exosomes. This development marks the official shift of the field from basic research to commercialization (2010–2025). As we advance, critical issues such as standardizing production, ensuring quality control, and understanding mechanisms of action need further attention (Figure 1).

### 1.2. Compositional Characteristics, Structural Advantages, and Functional Applications of Plant Exosomes

PDVs, also referred to as ELNs in many previous studies, have gained significant attention as natural nanocarriers because of their dual bioactivity and drug-delivery capabilities [10]. Unlike animal exosomes, current evidence suggests that the production of PDVs is a widespread feature across the plant kingdom rather than being restricted to particular tissues or species. PDVs have been successfully isolated from plant juices, individual plant organs (including fruits, roots, leaves, flowers, and seeds), and even whole-plant materials. Plant EVs have been identified in numerous species, such as cotton (*Gossypium* spp.), carrot, *Oryza sativa*, *Arabidopsis thaliana*, sunflower, *Olea europaea*, and *Nicotiana benthamiana*. Among these sources, succulent fruits (e.g., grape, grapefruit (*Citrus* × *paradisi*), and lemon) and rhizomatous medicinal plants (e.g., ginger (*Zingiber officinale*) and ginseng) have been reported to be particularly rich in PDVs and therefore represent the most extensively investigated sources for vesicle isolation. Their high vesicle yield also facilitates large-scale production and downstream biomedical applications. Nevertheless, the abundance, molecular composition, and biological functions of PDVs vary considerably among different plant species, taxonomic groups, and organs, making the selection of appropriate source materials an important consideration for downstream applications.

Compared with animal exosomes, PDVs exhibit several advantages, including low immunogenicity, abundant natural resources, and minimal ethical concerns [16]. Their endogenous active compounds have been reported to exhibit significant pharmacological activities [17], such as anti-tumour therapy [18], immune regulation [19], and maintaining intestinal microecological balance [20]. In contrast to traditional plant extracts, PDVs notably demonstrate greater therapeutic efficacy. For instance, Qiu et al. [21] reported that fresh exosomes from *Rehmannia glutinosa* exhibit comprehensive therapeutic effects through multi-pathway regulation, which outperforms the traditional plant extract.

PDVs have a phospholipid bilayer structure that encapsulates proteins, RNA, and small active molecules. Lipids make up 30–50% of their primary structural component, consisting of phosphatidylcholine, phosphatidic acid, and ceramide, which determines their biodistribution and cellular targeting properties. Grape-derived ELNs specifically target intestinal stem cells via lipid components to activate the Wnt/β-catenin pathway for tissue repair [10], while grapefruit ELNs achieve targeted drug delivery to intestinal macrophages through phosphatidylcholine-mediated gut–liver migration [22]. The protein in their structure acts as a functional executor. Citrus ELNs have been reported to have transmembrane transport proteins that can enhance drug bioavailability across intestinal barriers [23]. RNA components of PDVs, especially miRNAs, exhibit potential for cross-species gene regulation under lipid membrane protection. This phenomenon was demonstrated in ginger ELNs containing aly-miR396a-5p, which inhibits SARS-CoV-2 replication [24]. Small molecules, including flavonoids and anthocyanins, though present in low concentrations, synergistically enhance efficacy, such as strawberry (*Fragaria* × *ananassa*) ELNs protecting cells from oxidative damage without cytotoxicity [25]. This sophisticated multicomponent system provides exceptional stability, targeted specificity, and biocompatibility for innovative drug-delivery applications.

### 1.3. Technical Bottlenecks and Research Significance of PDVs

PDVs, emerging natural carriers, have demonstrated superior broad prospects in the biomedical field [21]. However, their transition into clinical use faces several challenges, particularly technical bottlenecks related to isolation and purification. The main issue is that current methods struggle to achieve high yield and purity simultaneously. Unlike animal-derived exosomes, isolating PDVs is limited by the complex structure of the plant cell wall and interference from secondary metabolites like polyphenols and polysaccharides [26]. Currently, ultracentrifugation (UC) causes vesicle aggregation and loss [27], while polymer precipitation results in insufficient purity and low recovery rates. Additionally, because PDVs are derived from various plant sources such as fruit pulp, roots, seeds, plant juices, rhizomes, and dried medicinal materials, developing a single, standardized isolation and purification protocol is challenging. This lack of standardization hampers research reproducibility and delays clinical translation [28]. Although the MISEV2018 guidelines by the ISEV provide a basic framework for EV research [29], source-specific solutions for the unique aspects of plant-derived exosomes are still needed.

Reviews in this field over the past two years have predominantly focused on downstream functional analyses such as pharmacological effects and clinical applications, while upstream separation and purification techniques that form the foundation for subsequent research are often glossed over with only broad descriptions [26,30]. As Theel et al. [31] emphasized, establishing a toolkit for exosome separation and purification is crucial for ensuring the reliability of downstream analysis and clinical translation. Despite significant advances in exosome isolation techniques in recent years, systematic, context-adaptive method evaluation and organ-specific optimization remain underdeveloped. Therefore, this paper systematically reevaluates current mainstream isolation and purification techniques from six practical dimensions. It also proposes innovative plant organ-oriented isolation strategies based on the physiological characteristics of different plant organs, providing a usage guide for the plant exosome isolation and purification toolkit.

## 2. Overview of PDV Separation and Purification Technology

### 2.1. Traditional Separation Technology

#### 2.1.1. Differential Ultracentrifugation and Density Gradient Centrifugation

Differential ultracentrifugation (DUC) is currently the most commonly used mainstream technology for the separation of PDVs. It operates on the principle of differences in the sedimentation coefficients of particles of varying sizes under centrifugal force. This method is popular because of its operational simplicity, high biocompatibility, and minimal equipment requirements. It is particularly effective for the initial separation of large-volume samples [32]. The standard procedure involves an initial low-speed centrifugation (500–10,000× *g*) to remove impurities such as cell debris, followed by high-speed centrifugation at 40,000–100,000× *g* to selectively precipitate PDVs. However, Bahri et al. [33] observed during their study of ginger-derived PDVs that although DUC can yield exosomes with higher purity, its extraction efficiency remains relatively low and depends heavily on high-precision centrifugation equipment. These limitations have somewhat restricted its use in large-scale production.

The physical properties of PDVs are heavily influenced by the structure of the plant cell wall and its diverse secondary metabolites. This variability is directly reflected in key parameters such as particle size distribution, yield, and purity after centrifugation, with differences far surpassing those seen in mammalian exosomes [34]. To address this challenge, researchers suggest optimizing factors like centrifugation duration, relative centrifugal force, and temperature to develop species-specific protocols [35]. Additionally, multiple centrifugation steps can easily cause structural damage to exosomes. Adding a high-density isotonic buffer layer, such as a sucrose cushion, at the bottom of the centrifuge tube can effectively preserve PDV structure and prevent aggregation [36]. Furthermore, using intermittent centrifugation strategies can improve precipitation efficiency and maintain structure by reducing membrane damage caused by sudden high-g-force exposure [37].

To further improve separation purity, DUC is often combined with density gradient centrifugation (DGC). This method allows precise particle isolation based on density differences by creating a descending density gradient of media [36]. Common gradient media include sucrose, iodixanol, and cesium chloride. Sucrose or iodixanol gradients primarily separate particles based on size and shape, whereas cesium chloride gradients rely on density differences [38]. Studies have shown that DGC is more effective than DUC in resolving complex samples, such as removing co-precipitated viral particles [39]. Although DGC offers high separation efficiency and minimal deformation of exosomes, it is important to consider that the gradient medium may affect subsequent experiments.

#### 2.1.2. Polymer Precipitation Method

Polymer precipitation has gained significant attention due to its ease of operation and high yield in PDV isolation. It uses polyethylene glycol (PEG) as a co-precipitant by decreasing the solubility of PDVs [40]. Studies confirm that PEG-precipitated vesicles, such as those from ginger, exhibit similar recovery rates and biochemical properties as their DUC-derived counterparts [41]. Besides PEG, other molecules such as protamine and sodium acetate can also precipitate charged EVs. However, co-precipitation of contaminating proteins remains a concern [42]. Savci et al. [43] optimized a PEG/dexamethasone two-phase system for separating PDVs from grapefruit juice, which exhibited enhanced impurity removal and purity. However, challenges persist due to interference from plant polysaccharides. Notably, precipitation methods are rarely used alone, with most researchers incorporating additional purification steps [44]. Recent advances, like pH adjustment and regulating EV surface charge, have significantly improved extraction efficiency. Bahri et al. [33] reported a fivefold increase in ginger PDVs yield under acidic conditions, highlighting the potential for optimized precipitation strategies.

#### 2.1.3. Size Exclusion Chromatography

Size-exclusion chromatography (SEC) is an emerging technique for EV separation, distinguished by its unique separation mechanism. The main principle is to accurately differentiate EVs from small molecular impurities, such as soluble proteins and protein aggregates, based on differences in the hydrodynamic radius of particles. This allows the use of chromatographic columns to effectively separate EVs in various biological fluids [45]. SEC is popular because it is easy to operate, prevents co-isolation of contaminants, and is scalable [46]. Results show that PDVs separated by SEC are not only extremely pure but also retain their vesicle structure and bioactive components, making them ideal for subsequent omics analysis and functional studies [47]. When extracting PDVs from cabbage juice, You et al. [48] compared SEC, ultrafiltration (UF), PEG precipitation, and DUC. They found that although UF is effective at removing large particle impurities, SEC can further separate nanovesicles from small molecular impurities like proteins, resulting in nanovesicles with much higher purity than those obtained by PEG precipitation and DUC. Additionally, their size distribution was the most uniform.

However, SEC separations face challenges. Cell wall-derived polysaccharides, such as pectin and cellulose, are very prone to causing column clogging, which leads to increased column pressure and decreased separation efficiency. To solve this problem, plant samples are usually pretreated with methods like enzymatic hydrolysis, UF, or affinity chromatography to remove polysaccharides before loading them into SEC. Alternatively, hydrophilic modified columns that resist clogging from polysaccharides are used to ensure stronger separations. Although SEC provides excellent separation performance, it takes longer to process. Because of this, it is often used as a polishing step along with UC or UF. This combined approach not only speeds up throughput but also effectively reduces the problem of polysaccharide clogging.

### 2.2. Emerging Separation Technologies

While traditional separation methods, such as DUC and PEG precipitation, have long been used in plant exosome isolation and have accumulated extensive experience, they have gradually exposed numerous limitations due to the unique properties of plant samples. These inherent limitations make it difficult for them to meet the diverse research demands of today, including high-purity and precise capture, scalable preparation, and active retention. In this predicament, the development and iterative upgrades of emerging separation technologies have become crucial for overcoming existing bottlenecks and advancing in-depth research on PDVs.

Currently, emerging PDV isolation technologies can be broadly classified from two complementary perspectives: (a) their primary design objective and (b) their technological development pathway. Technologies in the first category are developed to address specific unmet needs in PDV research, such as subtype-specific isolation or selective capture, and are therefore referred to as purpose-oriented technologies. In contrast, the second category comprises approaches that evolve from conventional isolation methods through technical optimization or integration to improve separation efficiency, scalability, automation, or vesicle preservation. Although some emerging technologies may possess characteristics of both categories, they are classified here according to their predominant design rationale and principal application. Based on this classification framework, this section discusses representative technologies within each category, focusing on their working principles, technical characteristics, advantages, limitations, and potential applications in PDV isolation.

#### 2.2.1. Specific Purpose-Oriented Technology

##### Immunoaffinity Method

**Figure 2:**
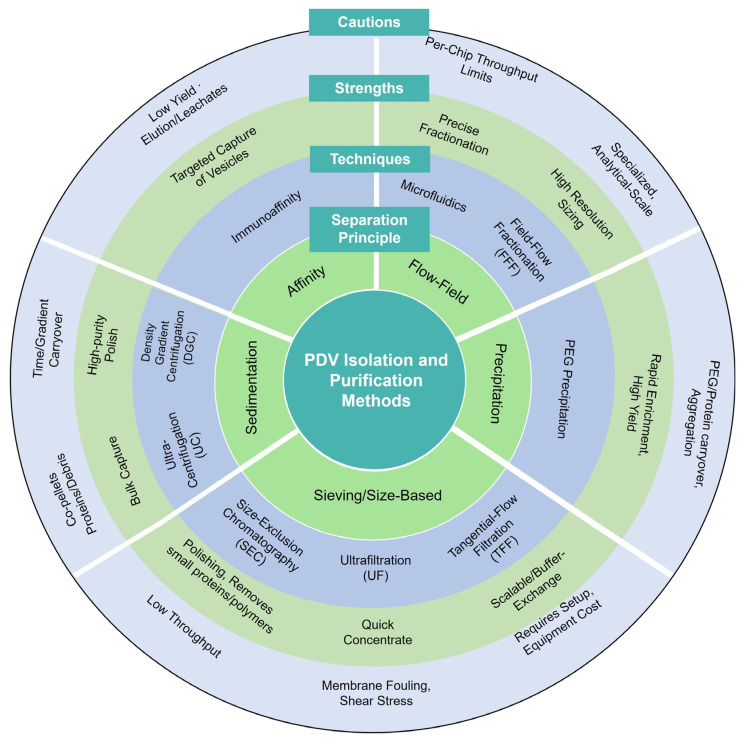
Isolation and purification landscape for PDVs. Concentric rings summarize PDV isolation approaches: inner ring—separation principles (sedimentation, precipitation, size-based sieving, affinity, flow-field); middle ring—representative techniques (UC, DGC, PEG precipitation, UF, TFF, SEC, immunoaffinity, microfluidics, AF4/FFF); outer ring—characteristic strengths and cautions for each technique (e.g., rapid enrichment or high-purity polishing vs. potential protein/PEG carry-over, shear/fouling, or gradient/time demands). The diagram serves as a qualitative guide for selecting PDV workflows by objective.

Immunoaffinity separation technology depends on specific antigen–antibody interactions that target vesicle surface markers to achieve high-purity isolation. This method features relatively simple procedures, with a single cycle completed effectively within 2–4 h [49]. Due to the lack of identified specific surface markers, its application in isolating plant exosomes remains limited. Studies have shown significant differences between the proteomes of plant and animal exosomes, making mammalian markers inapplicable for plant vesicle isolation [11]. Although some studies, like Huang et al., [50], have attempted to develop immunoaffinity strategies based on plant-specific proteins, successfully purifying TET8-positive exosome subtypes in Arabidopsis using antibodies targeting the EC2 domain of the TET8 protein, its potential for general use is limited by the absence of widely conserved markers. Currently, this method is more suitable for precise identification of plant exosomes rather than large-scale preparation. Resolving marker and antibody challenges in the future could enable the development of high-purity therapeutic ELNs (Figure 2).

#### 2.2.2. Improved Traditional Technologies

##### Microfluidics

Microfluidics, which utilizes microscale channels and chambers fabricated on a chip, enables the precise separation of exosomes. This technology effectively separates exosomes based on their physical properties, such as size, charge, and specific chemical interactions. It not only provides high-throughput separation and purification but also shows significant potential for automation. Addressing the issue that traditional centrifugation-based extraction is time-consuming and relies heavily on equipment such as centrifuges, pipettes, and tubes, which limits plant exosome extraction efficiency, Li et al. [51] proposed an innovative solution: designing a microfluidic chip for plant exosome extraction. This chip can collect plant exosomes of various sizes in multiple filter chambers and perform graded extraction of different-sized exosomes. This breakthrough significantly reduces dependence on centrifuges, shortens the experimental cycle, and dramatically enhances plant exosome extraction efficiency.

##### Field Flow Fractionation

Field flow fractionation is a non-stationary phase separation technology. Its principle involves applying an external force field perpendicular to the flow direction of the sample fluid in a flat channel. Because components of different particle sizes and molecular weights have different migration rates due to force differences, this allows for precise separation. In PDVs separation, field flow fractionation technology effectively addresses issues caused by stationary phase adsorption and the mechanical forces that damage vesicle integrity in traditional separation techniques. It benefits from characteristics such as no stationary phase, low system pressure, and minimal or no shear effects. The separation process minimally interferes with the structure and activity of plant ELNs [52]. Wu et al. [53] systematically optimized the core parameters of AF4, breaking through the technical bottleneck of “EVs and lipoprotein separation, for separating human plasma EVs”. They obtained high-purity, highly reproducible, and high-quality exosomes, thereby solving problems related to lipoprotein contamination, mechanical disruption of vesicle integrity, and the impact on the accuracy of omics results.

## 3. Methodological Performance Evaluation and Plant Organ-Directed Isolation Strategies

Choosing proper exosome isolation and purification methods is essential for ensuring the accuracy and reliability of subsequent research. This article systematically evaluates and recommends plant exosome isolation techniques from two main perspectives, aiming to give researchers a solid scientific foundation for selecting the most suitable method based on their specific needs.

### 3.1. Methodological Performance Evaluation Model Based on Six Criteria

ISEV emphasizes that when selecting an EV isolation method, researchers should fully consider its compatibility with the intended application. ISEV recommends categorizing EV isolation goals into four categories: high recovery with low specificity, moderate recovery with moderate specificity, low recovery with high specificity, and high recovery with high specificity. Based on this, mainstream isolation methods can be systematically evaluated based on six dimensions: operational difficulty, cost, throughput, purity, yield, and impact on vesicle integrity, which collectively reflect methodological feasibility, separation performance, scalability, and preservation of vesicle integrity (Table 1). The evaluation scores presented in Table 1 were assigned based on a comprehensive assessment of representative studies and methodological reviews using a literature-based semi-quantitative scoring approach. Researchers can assign different weights to these dimensions based on their research objectives to select the most appropriate isolation method. Because the performance of each isolation method may vary with sample type, experimental conditions, and downstream applications, this evaluation framework is intended to provide comparative guidance for method selection rather than absolute quantitative criteria.

### 3.2. Plant Organ-Oriented Separation Strategies

Isolating extracellular vesicles from plants requires tailored, organ-specific techniques given the complexity and diversity of plant tissues. Different plant organs have significant variations in their cellular structures, metabolite composition, and physicochemical properties. This makes PDV separation difficult when using a single standard process. This section systematically elaborates optimized separation strategies for different organs, including leaves, fruits, flowers, stems, and roots (Table 2).

#### 3.2.1. Sample Preparation Before PDVs Isolation

Prior to the formal isolation of PDVs, appropriate sample preparation is essential for releasing or enriching vesicles from plant materials and represents a critical prerequisite for obtaining high-quality PDVs. In general, mechanical disruption of plant tissues using methods such as grinding, squeezing, or blending represents the most fundamental and commonly used approach for releasing vesicle-containing fluids from different plant organs, including fruits and stems [83,84,85]. Although no studies have systematically compared the effects of different juice extraction methods on PDV isolation, previous studies have demonstrated that different sample processing strategies can significantly influence the phytochemical composition and biological activity of plant juices. For example, enzymatic and thermal treatments have been shown to alter the levels of phenolic compounds, amino acids, and volatile organic compounds in asparagus juice [86], suggesting that sample preparation may likewise influence the yield, composition, and biological activity of isolated PDVs. Moreover, because different plant organs exhibit substantial variations in tissue architecture, extracellular matrix composition, water content, and metabolite profiles, pretreatment strategies should be optimized according to the characteristics of the source material. Mechanical juice extraction inevitably disrupts plant tissues, leading to the co-extraction of extracellular matrix components, such as pectin, polysaccharides, and other impurities, which may compromise PDV purity. Therefore, appropriate pretreatment before vesicle isolation is essential for improving both vesicle recovery and purity. For instance, Tris-HCl is commonly added during citrus juice pretreatment to remove co-purified pectin [87]. Similarly, enzymatic digestion using cellulase has been applied to cellulose-rich plant tissues. Compared with direct UC, cellulase-assisted extraction of *Aloe vera* significantly improves extracellular vesicle recovery and purity, while reducing co-isolated contaminants and producing vesicles with a more homogeneous size distribution [88].

In addition to conventional juice extraction, PDVs can also be obtained from apoplastic washing fluid collected by vacuum infiltration, particularly for tissues with low juice content [89]. Furthermore, plant suspension-cultured cells and callus cultures have emerged as alternative PDV sources [90]. These alternative preparation strategies further expand the range of available PDV sources and provide greater flexibility for downstream isolation and large-scale production. As PDV research continues to advance, the development of gentler, more standardized, and highly reproducible sample preparation strategies is expected to further improve PDV purity, recovery, and batch-to-batch consistency, thereby providing a reliable foundation for subsequent isolation, characterization, and functional studies.

#### 3.2.2. Leaves

For leaves with a general structure, DUC is a more suitable technique due to its operational simplicity. It effectively removes large particulate impurities, such as chloroplast fragments, and achieves preliminary vesicle enrichment. However, leaves with special structures, such as those of Apocynaceae plants, their multiple epidermis, thick cuticle, high pectin content, and laticifer networks form physical barriers that severely hinder vesicle release. To overcome this challenge, Ou et al. [74] proposed a strategy combining enzymatic treatment (e.g., pectinase and cellulase) with DUC. This technique effectively deconstructed the cell wall network and improved vesicle yield. When higher vesicle purity is required, DGC can provide purer products but faces limitations of low yield and long processing time. In contrast, UF combined with SEC effectively removes contaminants while maintaining good yield. It has proven to be a reliable method for obtaining leaf-derived vesicles with high homogeneity [48].

#### 3.2.3. Fruits

Fruit tissues can be broadly classified into two categories based on their physicochemical properties: low-viscosity, low-sugar fruits and high-viscosity, high-sugar fruits. For low-viscosity, low-sugar fruits, DUC has become a popular separation method due to its ease of use and low cost [91,92]. However, for fruits with high pectin content, like blueberries (*Vaccinium* spp.), the separation process is more difficult. Pectin easily forms gel complexes with vesicles under the high centrifugal force of UC, making it harder to isolate exosomes effectively [93]. To overcome this challenge, Iriawati et al. [94] suggested pretreating samples with pectinase to disrupt the gel network, followed by using PEG for enrichment. This approach effectively overcomes the extraction challenge caused by high pectin content. For fruit tissues with high free sugar content, such as pomegranate, high osmotic pressure causes vesicle structure to collapse. To tackle this issue effectively, tangential flow ultrafiltration combined with SEC was used [47]. This combined approach not only alleviates the osmotic stress caused by high sugar levels but also preserves the integrity and functional activity of the vesicles.

#### 3.2.4. Flowers

In floral tissues, exosome isolation is hindered by polysaccharides, non-exosomal vesicles, and abundant pigments. DUC serves as a preliminary purification step, while sucrose DGC enables more precise separation based on density differences. It effectively removes co-purified contaminants [78]. Li et al. [94] demonstrated an effective protocol for isolating honeysuckle (*Lonicera japonica*)-derived nanovesicles (HNVs) from dried flowers of honeysuckle. After soaking dried flowers in phosphate-buffered saline and homogenizing the tissue, repeated 10,000 rpm centrifugation was applied to clarify the sample. The supernatants were then processed through hollow-fiber tangential flow filtration (200 nm pore size, 750 kDa MWCO) to separate vesicles ≤ 200 nm, which were collected as HNVs. This method resulted in the recovery of nanovesicles with a narrow size distribution, a negative zeta potential, and stable morphology, confirmed by cryo-electron microscopy (Cryo-EM). Notably, the HNVs retained their integrity and functionality when exposed to simulated gastric and intestinal fluids, making them suitable for oral administration.

#### 3.2.5. Stems

Currently, most research on exosomes from plant stems focuses on modified stems, such as rhizomes (ginger and *Curcuma longa*), tubers (*Dioscorea opposita* and *Solanum tuberosum*), and bulbs (*Allium cepa* and *Allium sativum*). These modified stems primarily serve as nutrient storage in the original plant and are therefore rich in starch, protein, polysaccharides, and flavonoids, which exhibit high viscosity and significant impurities [95,96]. DUC combined with sucrose DGC unique advantages for such materials, particularly excelling in the retention of active components [97].

#### 3.2.6. Roots

Root tissues have complex compositions, featuring tightly packed cells that contain numerous starch particles and secondary metabolites similar in size to exosomes. These factors present numerous challenges to isolating exosomes. To address these issues, combining DUC with sucrose DGC effectively removes large particle impurities and some cell fragments. It also separates impurities similar in size to exosomes, such as small starch granules and mitochondrial fragments, based on density differences. This combined approach allows for the collection of high-quality, pure exosome materials, laying a solid foundation for subsequent biological research and applications [98,99].

## 4. Quality Control and Standardized Characterization of PDVs

In a study on methods for separating and purifying ELNs from *Houttuynia cordata*, Cong et al. [100] found that the antibacterial activity of ELNs isolated using the “low-speed centrifugation combined with UF” method was significantly higher than that of products separated by the traditional differential centrifugation method. This phenomenon is not coincidental. Studies have confirmed that the functional activity of PDVs is closely linked to their source species and preparation process. PDVs from different separation methods or plant sources not only show significant activity differences but may also have opposite effects. These research findings demonstrate that the choice of separation and purification method critically influences the activity and safety of exosomes [101].

**Figure 3:**
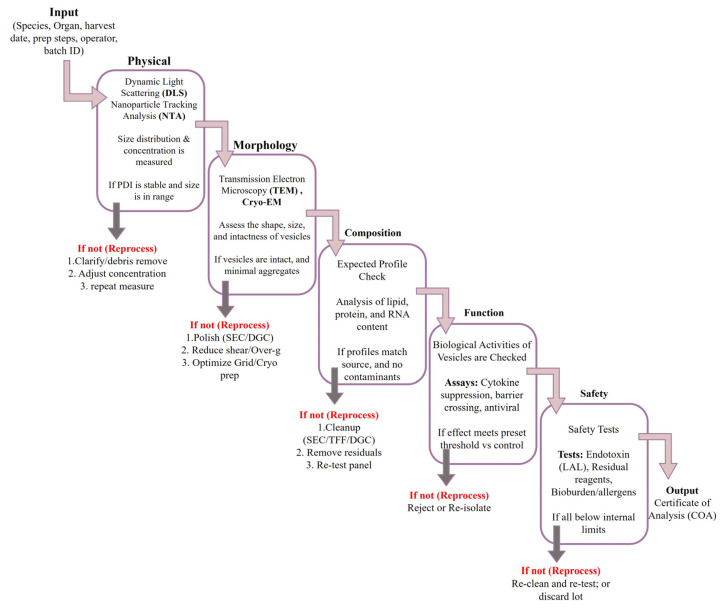
Quality-control and standardized batch-release pipeline for PDVs. Batches enter with defined metadata (species/tissue, process log/SOP, batch ID, dates, starting mass or volume, storage conditions). QC then proceeds through a series of Go/No-Go gates. Physical testing uses DLS/NTA to assess hydrodynamic size distribution and particle concentration. Morphology is examined by TEM or cryo-EM (±AFM) to verify vesicle integrity and aggregation state. Composition profiling quantifies lipids, proteins, RNAs, and metabolites, including the particle-to-protein ratio and checks for residual PEG/solvents. Function is evaluated in fit-for-purpose bioassays (e.g., cytokine suppression, barrier crossing, antiviral activity). Safety testing includes endotoxin (LAL), residual reagents, and bioburden/allergens as applicable. Failure at any gate triggers corrective processing and re-testing (or rejection); lots that pass all gates receive a Certificate of Analysis documenting results and release status.

However, there is still a lack of unified standards and methods for the quality control and standardized characterization of plant exosomes. Accurately assessing the purity, integrity, and biological activity of exosomes is an urgent issue that requires resolution. At the same time, there is no consensus on how to standardize the characterization of plant exosomes, which makes it difficult to compare and reproduce results across different research teams and severely hinders their clinical development. Therefore, establishing standardized characterization methods and quality control systems is essential for ensuring the safety and effectiveness of plant exosomes in clinical use and for advancing from laboratory research to clinical application (Figure 3).

### 4.1. Physical Characterization

Physical characterization represents the first step in PDV identification and quality evaluation. Because PDVs are heterogeneous nanoscale vesicles containing diverse molecular cargos, comprehensive physical characterization is essential to confirm vesicle identity, evaluate sample purity, assess structural integrity, and ensure reproducibility between preparation batches. Key physical parameters include particle size distribution, particle concentration, morphology, and surface charge. These variations are closely linked to plant cell type, growth stage physiology, and the efficiency of enriching particles of different sizes during extraction and purification [102,103].

#### 4.1.1. Particle Size Distribution and Concentration

Particle size distribution is the primary physical parameter for distinguishing different EV subpopulations. Accurate determination of particle size distribution is a prerequisite for reliable biochemical characterization and functional evaluation, as heterogeneous vesicle populations may interfere with downstream analyses [104]. Moreover, particle size is closely associated with molecular composition. Previous studies have demonstrated that vesicle diameter determines the relative proportion of membrane-associated proteins and intraluminal proteins, indicating that particle size directly influences the molecular composition of EVs and thereby affects their potential biological functions [105]. Consistent with this, different size fractions of glioma stem cell-derived exosomes have been shown to exhibit distinct cellular uptake efficiencies and biological activities [106]. While particle size describes the physical characteristics of PDVs, particle concentration provides the most fundamental quantitative measure of a PDV preparation by indicating the number of vesicles present in a sample. This parameter serves as a direct basis for determining the dosage used in subsequent in vitro and in vivo experiments [107]. The combined assessment of particle size and concentration also facilitates meaningful comparisons among different studies, thereby promoting the standardization and reproducibility of PDV research.

For measuring the particle size and concentration of ELNs, dynamic light scattering (DLS) and nanoparticle tracking analysis (NTA) are the two most commonly used techniques. DLS relies on autocorrelation analysis of fluctuations in laser-scattered light intensity caused by the Brownian motion of particles, producing the intensity-weighted hydrodynamic diameter (Z-average). In addition, DLS can determine the polydispersity index (PDI), which reflects the homogeneity of the particle population, with lower PDI values generally indicating a more uniform sample. It provides quick analysis, but its resolution is low and it is highly sensitive to the sample’s scattering properties. Consequently, DLS is well suited for rapid assessment of the average particle size and overall sample uniformity, although it may overestimate particle size in heterogeneous samples or in the presence of aggregates [108]. However, because DLS derives particle size from ensemble light-scattering signals, complementary techniques are often required to achieve more accurate characterization of heterogeneous PDV populations. In contrast, NTA measures particle size and concentration by tracking the Brownian motion of individual particles. Unlike DLS, which analyzes the ensemble scattering signal, NTA characterizes individual vesicles and therefore provides both particle size distribution and absolute particle concentration. These measurements enable quantitative evaluation of vesicle abundance and sample heterogeneity, providing an important basis for dosage normalization and subsequent functional studies. It has higher resolution than DLS and is less affected by the strong scattering of larger particles, making it especially suitable for polydisperse systems [109,110]. Because DLS rapidly determines the average hydrodynamic diameter and sample homogeneity, whereas NTA simultaneously provides particle size distribution and concentration at the single-particle level, the combined use of these two techniques enables a more comprehensive evaluation of PDV size characteristics and preparation quality. Combining DLS and NTA is therefore recommended to ensure accurate analysis [111].

#### 4.1.2. Morphology

Morphological characterization provides direct evidence for the identification and quality evaluation of PDVs. PDVs possess characteristic morphological features and are commonly observed as cup-shaped or saucer-like vesicles after conventional sample preparation. Therefore, morphological characterization serves as one of the important criteria for distinguishing PDVs from non-vesicular particles and contaminants. Furthermore, intact vesicles with well-preserved membrane structures generally indicate high-quality PDV preparations and suggest that the isolation and purification procedures effectively preserve vesicle integrity while minimizing structural damage. In contrast, collapsed, disrupted, or aggregated vesicles may indicate sample degradation or inappropriate isolation conditions.

In morphological characterization, transmission electron microscopy (TEM) is widely used to visualize the morphology, size, and membrane integrity of PDVs at the nanoscale. TEM generates high-resolution images by transmitting electrons through ultrathin samples, enabling direct observation of vesicle shape, membrane boundaries, and structural integrity. However, conventional TEM preparation involving dehydration and fixation may cause structural deformation. In addition, plant-derived polysaccharides may generate “polysaccharide artifacts”, including aggregation, crystallization, and carbonization of sugars during vacuum drying or high-energy electron beam exposure. To overcome these limitations and obtain more accurate structural information, Cryo-EM has increasingly been adopted for PDV characterization. Cryo-EM enables direct visualization of PDVs under cryogenic conditions by rapidly freezing samples to preserve their native hydrated state. Compared with conventional TEM, Cryo-EM minimizes dehydration- and fixation-induced deformation and provides more accurate information regarding vesicle morphology, membrane architecture, and structural integrity [112,113]. In addition to electron microscopy-based techniques, atomic force microscopy (AFM) provides complementary structural information by characterizing the three-dimensional surface topography and mechanical properties of PDVs. AFM provides three-dimensional surface imaging by scanning samples with a nanoscale probe and detecting interactions between the probe and the sample surface. In addition to topographical information, AFM enables measurement of mechanical properties, including membrane stiffness, rigidity, and elasticity, without requiring extensive sample preparation. These structural and mechanical parameters provide insights into vesicle stability, membrane properties, and potential interactions with recipient cells, making AFM valuable for investigating the biological functions and delivery potential of PDVs [114,115].

#### 4.1.3. Surface Charge

Surface charge is an important indicator of PDV colloidal stability. The zeta potential reflects the electrostatic repulsion between vesicles, which prevents aggregation and maintains dispersion stability. Studies have shown that the surface potential of plant-derived vesicles generally ranges from near neutral to approximately 50 mV [116]. Higher absolute zeta potential values indicate stronger repulsive forces and greater colloidal stability, whereas reduced surface charge may increase the tendency of vesicle aggregation. To quantitatively evaluate these surface charge characteristics, electrophoretic light scattering is the most commonly used analytical technique. It determines the surface charge properties of PDVs by measuring the electrophoretic mobility of charged vesicles under an applied electric field. The resulting zeta potential values provide information on vesicle surface characteristics and are commonly used to assess the stability and quality of PDV preparations.

### 4.2. Component Analysis and Functional Activity Evaluation

The clinical translation of PDV necessitates a thorough understanding of its “chemical composition–structure–bioactivity” relationship. Systematic profiling of composition and functional validation are fundamental for evaluating the biomedical potential and safety profiles of PDVs. The functional activities of PDVs arise from their specific chemical constituents, including nucleic acids, functional proteins, structural lipids, and plant-specific secondary metabolites, which collectively contribute to their structural characteristics and biological activities. Therefore, comprehensive characterization of these components is required to establish molecular fingerprints of PDVs and clarify the contribution of individual components to their functional effects.

#### 4.2.1. Component Analysis of PDVs

PDVs contain diverse molecular components, including nucleic acids, proteins, and plant-derived small molecules, which collectively determine their structural properties and biological functions. Therefore, systematic analysis of these components is essential for elucidating the molecular basis underlying PDV activity and facilitating their biomedical applications. In addition, compositional analysis must address both efficacy and safety, while identifying active components, it is equally important to detect and remove potential allergenic impurities or toxic residues from the isolation process, which is a prerequisite for clinical advancement [117,118]. In this section, the analytical approaches used for the characterization of different molecular components are discussed.

##### Nucleic Acid Analysis

Nucleic acids represent one of the most important functional cargos of PDVs and contribute substantially to their biological activities. PDVs are enriched in various RNA cargos, including miRNAs, mRNAs, and lncRNAs. Notably, miRNAs can regulate host gene expression through cross-species transfer, leading to biological activities such as anti-inflammatory, antiviral, and anti-fibrotic effects [119]. To characterize these RNA cargos and elucidate their potential biological functions, a variety of molecular analytical techniques have been developed. High-throughput sequencing technologies, including small RNA sequencing and RNA sequencing, are widely used to comprehensively characterize RNA cargos within PDVs [120]. These approaches identify different RNA species and provide information regarding RNA diversity, abundance, and potential regulatory functions. For specific RNA molecules, quantitative techniques such as quantitative real-time PCR and droplet digital PCR enable sensitive detection and accurate quantification, allowing further validation of sequencing results and assessment of RNA enrichment levels [121,122]. Together, these approaches provide important molecular evidence for understanding how PDV-derived RNAs contribute to biological regulation and therapeutic effects.

##### Protein Analysis

Proteins represent essential components of PDVs and play important roles in vesicle formation, membrane stability, cellular recognition, and biological regulation [123,124]. Analysis of protein composition can not only reveal the overall protein landscape of PDVs but also help identify specific proteins associated with vesicle biogenesis, targeting ability, and biological activities. Sodium dodecyl sulfate–polyacrylamide gel electrophoresis (SDS-PAGE) is commonly used as a preliminary approach for protein characterization. Based on the separation of proteins according to their molecular weight, SDS-PAGE provides an overview of protein distribution patterns and allows rapid comparison of protein profiles among different PDV preparations. However, this method provides limited information regarding protein identity and function [125]. Mass spectrometry-based proteomic approaches, by contrast, provide more comprehensive protein characterization by identifying and quantifying individual proteins based on their specific peptide signatures. These approaches enable systematic analysis of protein composition within PDVs and facilitate the discovery of functional proteins involved in vesicle stability, cellular uptake, molecular targeting, and biological regulation [126,127]. Therefore, integrating SDS-PAGE with advanced proteomic techniques can provide complementary information regarding both overall protein profiles and specific functional components, contributing to a deeper understanding of the relationship between PDV protein composition and biological functions.

##### Metabolite Analysis

Plant-specific metabolites represent unique components of PDVs and contribute to their distinct biological properties. These bioactive molecules, including ginsenosides, gingerol, and β-sitosterol, have been identified in PDVs and may contribute to their therapeutic effects [128]. Accordingly, metabolomic approaches, particularly liquid chromatography coupled with tandem mass spectrometry, are widely used to identify and quantify metabolites within PDVs. These techniques characterize metabolites based on chromatographic separation, mass-to-charge ratios, and fragmentation patterns, providing information regarding metabolite composition, abundance, and variation among different PDV sources. Such analyses facilitate the identification of bioactive molecules responsible for PDV functions and help elucidate the contribution of plant-specific metabolites to vesicle biological activities [129,130].

#### 4.2.2. Functional Activity Evaluation

Functional validation acts as the essential link connecting compositional analysis to biological applications. While compositional analysis provides information regarding the potential bioactive components of PDVs, functional evaluation is necessary to verify whether these components contribute to specific biological effects. The biological activities of PDVs can be assessed using well-designed in vitro or in vivo models combined with relevant functional and molecular indicators. For example, after quantitatively identifying 26 ginsenosides in ginseng-derived ELNs, Li et al. [131] further demonstrated their notable anti-inflammatory effects. In an LPS-induced macrophage inflammation model, they monitored the expression levels of inflammatory mediators such as NO, TNF-α, and IL-6.

## 5. Challenges and Future Perspectives

**Figure 4:**
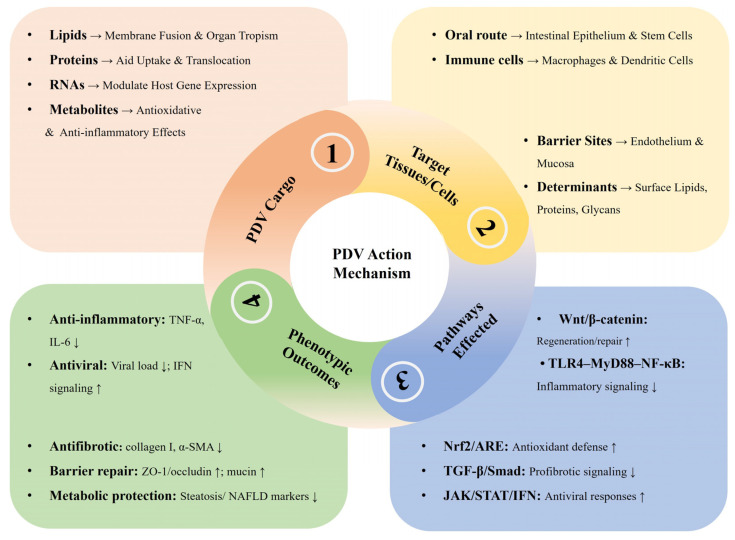
Mechanism-of-action map for PDVs. A four-step schematic action map is shown here. Cargo comprises lipids, proteins, RNAs, and metabolites, which provide bioactive signals and targeting determinants. Target tissues/cells are reached primarily via the oral route (intestinal epithelium and stem cells; macrophages/dendritic cells along the gut–liver axis; barrier endothelium/mucosa), with other routes used as indicated. Surface lipids, proteins, and glycans guide biodistribution and uptake. Key host pathways modulated include Wnt/β-catenin, TLR4–MyD88–NF-κB, and oxidative-stress pathways (e.g., Nrf2/ARE or AP-1/ROS). Phenotypic outcomes include anti-inflammatory, antiviral, antifibrotic, barrier repair, and metabolic protection effects. Arrows indication: ↑ increase; ↓ decrease.

The transition of PDVs from promising biological entities to clinically viable therapeutics faces complex challenges. PDVs have proven to be desirable candidates for therapeutics due to their loading capacity and potential bioactivity [10] (Figure 4). However, to realize their potential, key bottlenecks in standardization, characterization, and functional assessment must be systematically addressed. The main challenge lies in establishing robust and scalable manufacturing processes. Current isolation techniques (such as traditional UC, immunoaffinity methods, etc.) consistently face a fundamental trade-off between yield and purity, which limits their production for clinical applications [41,44]. Future innovations must address this issue by applying novel engineering solutions, such as microfluidic systems and advanced filtration, to enable large-scale production and the reproducibility of PDVs [47,131]. In parallel, a deeper understanding of PDV biology is essential. The current absence of universal or tissue-specific biomarkers severely hinders accurate identification and functional analysis [11,50]. A systematic multi-omics framework should be established by integrating lipidomics, proteomics, and transcriptomics to decode the complete compositional spectrum of PDVs [121,132].

Furthermore, the transition of PDVs from laboratories to clinical applications is constrained by the lack of standardized techniques for evaluating their biological potency and determining their dosing. Reported effective doses across studies vary widely because the assigned doses are derived from different PDV sources and animal models. This impedes meaningful comparisons and the clinical translation of PDVS [12,13]. Finally, strengthening interdisciplinary collaboration is also crucial. The synergy of multi-omics technology and bioinformatics has opened new pathways for systematically characterizing the full spectrum of PDVs. Using advanced algorithms, essential information in large datasets can be accurately extracted to build a detailed and comprehensive database, clarifying the functional roles and interaction mechanisms of each component. Integrating chemical engineering and materials science not only advances the development of new separation materials and processes to improve the recovery rate and purity of PDVs, but also emphasizes innovative delivery system designs that enhance their stability and targeting in vivo, laying a solid foundation for clinical application. The deep integration of artificial intelligence (AI) and automation technologies has transformed the PDVs research process—from precisely optimizing extraction parameters and predicting biological activity to designing experiments intelligently and achieving full-process automation from sample processing to data analysis. This significantly increases research efficiency, improves result reproducibility, and provides robust support for scientific discovery. Interdisciplinary collaboration is key to ushering in a new era in PDVs research. It not only injects innovative vitality into current studies but also paves the way for widespread clinical use of PDVs in the future. This will elevate this cutting-edge field, enabling the efficient translation of scientific discoveries into clinical practice and offering new hope for disease treatment.

In conclusion, the path toward clinical adoption of PDVs, though demanding, is clearly delineated. A concerted focus on standardizing manufacturing, elucidating biological mechanisms, and establishing efficacy-driven evaluation frameworks will be pivotal to transforming PDVs from a scientific curiosity into a new class of versatile, practical clinical therapeutics, ultimately fulfilling their potential to address unmet medical needs.

## Figures and Tables

**Table 1:** Methodological Performance Evaluation of PDVs Based on Six Criteria.

Evaluation Dimension	Coprecipitation Method		Ultrafiltration		Size Exclusion Chromatography		Immunoaffinity Method		Differential Ultracentrifugation		Density Gradient Centrifugation	
	Description	Score	Description	Score	Description	Score	Description	Score	Description	Score	Description	Score
Operation Difficulty	Low operation difficulty	5	Simple procedure, but membrane pore size selection and clogging prevention need control	4	Need to balance eluent flow rate, column temperature and other parameters, rely on chromatograph operation skills, low process standardization	1	Simple procedure and automatable (e.g., magnetic bead sorting)	4	Requires multiple centrifugation steps, different centrifugation steps need to set different speeds and times, certain experience required	3	Based on differential ultracentrifugation, accurate configuration of different density media is needed, high operational complexity	2
Cost (Equipment + Reagents)	Low cost	5	High equipment cost, high membrane replacement frequency	2	High cost	1	Extremely high cost, high cost of specific antibodies, large carrier consumption	1	Need to purchase ultracentrifuge, high equipment cost	2	Relatively high cost	2
Throughput	Large volume samples can be processed at one time, no obvious volume limitation	5	Tangential flow is suitable for large-scale processing, conventional membrane tubes are suitable for small samples	3	Extremely small single processing volume, limited column capacity, not suitable for batch samples	1	Extremely small throughput, limited antibody carrier load	1	Medium single throughput, total throughput can be increased to a certain extent through batch processing	3	Relatively small single throughput	2
Purity	Low purity, easy to coprecipitate free proteins, polysaccharides and other impurities, difficult subsequent purification	1	Small molecules can be removed by membrane pore size interception	3	Relatively high purity	4	Extremely high purity, only target exosomes are captured through antigen–antibody specific binding, almost no impurity residue	5	Can remove most large particle impurities such as cell debris and dead cells, but it is difficult to completely remove impurities with density, size and shape similar to exosomes because separation only relies on particle density, size and shape	3	Rely on density separation, can more effectively separate exosomes from other impurities such as virus particles and lipoproteins	4
Yield	Efficient enrichment by reducing exosome solubility	5	Partial exosomes are lost due to adsorption on the membrane surface, yield is greatly affected by membrane material	3	Partial exosomes are lost due to adsorption on chromatographic column packing, yield is greatly affected by column type	3	Extremely low yield, only exosomes expressing specific markers can be captured, and antibody binding efficiency is limited	1	Operation often involves multiple centrifugations, with large residual loss. Incorrect setting of centrifugation conditions can easily lead to ineffective exosome precipitation	3	Some exosomes may interact with density gradient media or shift position during centrifugation, resulting in ineffective collection	3
Impact on Vesicle Integrity	Precipitants (e.g., high-concentration PEG) may slightly damage membrane structure, but no mechanical damage	4	No mechanical force from high-speed centrifugation, only driven through the membrane by pressure, low risk of membrane structure damage	5	Only mild elution, high integrity retention rate	5	Only mild elution to break the binding, good integrity retention	5	Large mechanical force is generated during high-speed centrifugation, exosome membrane structure is easily damaged	2	Exosomes gradually sediment and enrich in the density gradient medium, relatively small mechanical impact	3
Reference	[41,54]		[55,56,57]		[48,58]		[59,60,61]		[62,63,64]		[65,66]	

Footnote. Each method is scored for each criterion on a scale of 1 to 5, where a higher score indicates better overall performance.

**Table 2:** Recommended Guide for Plant Organ-Directed Isolation Strategies.

Organ	Organ Characteristics	Recommended Treatment	Remarks	Additional References
Leaves	General: Mainly contains larger particle impurities such as chloroplast fragments and cell wall residues	DUC	Can quickly and effectively remove large particle debris impurities	[67]
		DUC + DGC	Low yield, general vesicle integrity; relatively high purity	[68]
		UF + SEC	Effectively remove co-purified proteins, suitable for subsequent proteomics analysis	[69,70,71]
	Apocynaceae: The multiple epidermis, thick cuticle, high pectin content and laticifer network of leaves form a dual barrier of rigidity and viscosity	Pectinase/Cellulase + DUC	Enzyme treatment reduces viscosity and loosens the network structure, efficiently enriches exosomes	[72,73,74]
Fruit	Low viscosity, low sugar	DUC	Simple operation, low cost	[75]
	High pectin: Pectin forms a “gel-like complex” with vesicles and proteins, leading to difficulty in separation	Pectinase + DUC + PEG Precipitation	Enzyme treatment destroys the gel structure, centrifugation removes fiber impurities, and PEG enriches	[76]
	High free sugar	TFF + SEC	Can effectively relieve hyperosmotic stress, maintain vesicle structural integrity and functional activity	[77]
Flower	Contains a large amount of polysaccharides, non-exosomal vesicles overlapping with exosomes, and high-abundance pigments	DUC + DGC	UC followed by DGC (±TFF) helps deplete pigment-rich non-exosomal vesicles and provides high-purity, GI-stable flower-derived PDVs	[78,79]
Stem (Rhizome, Tuber, Bulb)	Rich in starch, protein, polysaccharides, flavonoids and other components, showing characteristics of high viscosity and many impurities	DUC + DGC	Can have both high purity and high activity	[80,81]
Root	Cells are closely arranged, and a large number of cell fragments are easily released after crushing; rich in carbohydrate storage substances and secondary metabolites	DUC + DGC	Can effectively remove large particle impurities and some cell debris, and further separate impurities similar in size to exosomes based on density differences	[82]

## Data Availability

No new data were created or analyzed in this study.

## Abbreviations

The following abbreviations are used in this manuscript:

PDV	plant-derived vesicles
ISEV	International Society for Extracellular Vesicles
ELN	exosome-like nanoparticle
UC	ultracentrifugation
DUC	differential ultracentrifugation
DGC	density gradient centrifugation
SEC	size-exclusion chromatography
UF	ultrafiltration
DLS	dynamic Light Scattering
NTA	nanoparticle tracking analysis
TEM	transmission electron microscopy
AFM	atomic force microscopy
PEG	polyethylene glycol
Cryo-EM	cryo-electron microscopy
SDS-PAGE	sodium dodecyl sulfate–polyacrylamide gel electrophoresis
